# A Study of the Perception of Health Risks among College Students in China

**DOI:** 10.3390/ijerph10062133

**Published:** 2013-05-27

**Authors:** Chenggang Zhang, Jingbo Fan

**Affiliations:** 1 School of Social Sciences, Tsinghua University, Beijing 100084, China; 2 College of Humanities & Social Sciences, University of Chinese Academy of Sciences, Beijing 100049, China

**Keywords:** health risk, risk perception, college students, geographic differences, gender differences

## Abstract

The present survey was designed to investigate the perception of health risks among college students in China. The data are the responses of a sample of 3,069 college students at one university to surveys that include measures of several dimensions of public judgments about fifteen specific hazards. Chinese college students conveyed their concerns as falling into three broad categories: Environmental (e.g., global warming, natural catastrophes, the ozone hole, air pollution, chemical pollution, pesticides in food), Technological (e.g., nuclear power stations, thermal power, genetically modified food, medical X-rays), and Social (cigarette smoking, drinking alcohol, overtime study or work, mental stress, motor vehicle accidents). The data were collected with a self-report questionnaire. Descriptive statistics were used to illustrate the levels of perceived risk according to the percent of “high risk” responses as well as the mean response values. Generally, the hazards that were perceived as posing the greatest health risk were those belonging to the social health risks; items related to technology risks received the lowest percentage of “high health risk” rankings. Traditional environmental risks such as natural catastrophes, pollution issues (chemical pollution, air pollution), and pesticides in food were ranked as being relatively high risks. The respondents were less concerned about new emerging issues and long-term environmental risks (global warming). In this survey, motor vehicle accidents were considered to be a “high health risk” by the greatest percentage of respondents. Generally speaking, the female respondents’ degree of recognition of health risks is higher than that of male respondents. Only for the item of smoking was the male respondents’ degree higher than that of females. There is also a geographic imbalance in the health risk perceptions. The degree of recognition of health risks from respondents in municipalities is generally lower than that of respondents from other areas except for items such as natural disasters, smoking, medical X-rays, and mental stress, which are exceptions.

## 1. Introduction

Since implementing reforms and a new policy of openness in 1978, China has emerged as an increasingly important player in today’s global economy and in international affairs and business. According to a joint report in 2012 by The World Meteorological Organization and the International Global Atmospheric Chemistry Project, the rapid development of urbanization will confront residents with more health risks. China is experiencing a high-speed urbanization process that will leave more people living with health risks.

People respond to hazards according to their perception of the risks that they pose. The public perception of risk is an important consideration in public health and risk management decision-making. The perceptions of risk vary with age, gender, education, and geographic region. Decision-makers need to consider how the public values risk issues. A better understanding of the factors shaping the public perception of health issues and risks will help to determine how the perception of risk should be addressed when managing health risk issues [1].

Risk perception is the focus of many current social science investigations and applications. The beginning of risk perception research can be traced to the nuclear debate of the 1960s. Researchers during this period showed that risk acceptance was related not only to the technical estimates of risk and benefits but also to a subjective dimension [2]. The ubiquity of risk and its prominence in the public sector has prompted a sustained research effort to understand how people perceive risks. Over the years, a collection of studies [3,4,5,6] has focused on identifying the factors that affect the perception of risk. These studies have consistently demonstrated that public perceptions reflect a vast array of considerations other than the actual risk. Previous research has identified five broad determinants of health: the physical environment, biology, lifestyle, the social environment and health care. Previous research has demonstrated that perceptions of health risk are influenced both by the qualities of the hazard and by the characteristics of the risk perceivers themselves. Among the factors linked to the hazards, the “dread factor” has been repeatedly shown to be the most determinant. Other factors linked to hazards have also been shown to influence risk perception: the perceived catastrophic potential of the hazard, the perceived lack of control over the situation, the unknown character of the hazard, and the number of people potentially affected.

Inspired by previous studies [7,8,9,10], the present study develops a grouping of health hazards: health-related environmental risks, health-related technological risks, and health-related social risks. Specifically, the hazards considered under the Environmental health risk perceptions reflected the physical environment; the environmental risk cluster included items relating to chemicals, contaminants or air pollution. The hazards examined in the Technological health risk perceptions reflected the technologies of health improvement and health care, such as medical X-rays. The hazards related to Social health risk perceptions reflected either lifestyle or the social environment. The examples included within Social risks were motor vehicle accidents, cigarette smoking, *etc.* However, some hazards, although few in number, affected more than one health risk component. For instance, genetically modified food (GMF) was primarily associated with Environmental health risk perception and to a lesser extent onto Social health risk because these foods entail a certain degree of personal choice.

Most previous studies have focused on the following specific health risk issues: (1) Environmental health risks: ecological risk [11,12], global warming [13], air pollution [14], nuclear power [15,16,17]; (2) Technological health risks: medical X-rays [18,19], GM foods [20]; (3) Social health risks: alcohol use [21]; motor vehicle accidents [22,23]. Scholars have proposed different important variables to understand how people view risks and their ability to reduce threats [24,25,26,27,28]. The attitudes regarding GMF among Korean and U.S. college students was reported; 4,231 people from 17 cities in China were involved in a stratified sampling survey to explore perceptions of SARS using the socio-psychological predictive model; HIV/AIDS knowledge, attitudes, and perceptions and personal risk behaviors among undergraduate students in China were described; people’s risk beliefs and policy attitudes about climate change were assessed; and the differences in HIV knowledge and attitude between gender and school years among college students in China was explored. The risk perceptions in Northeast Asia were investigated [29], and the health-related quality of life and work-related stress and its risk factors among white-collar businessmen and management workers were examined [30].

The perception of health risk among the Chinese has seldom been studied. Typically, the data from other countries are used to determine what hazards might be considered to be most or the least risky by the public. However, cultural and social context may influence public health risk perceptions. There have been few studies on the general health risk perception of college students, but even less research is available that concentrates on the health risk perceptions among Chinese college students. Most previous work has been conducted with convenience samples or with samples that have very low response rates [31]; in the present paper, results from a large representative sample of the Chinese college students is presented.

College students are considered to be emerging adults, a developmental period that is characterized as an age of identity exploration, instability, and of feeling “in-between” [32]. This article focuses on a descriptive account of the ratings of the perceived risk from fifteen specific hazards in a representative sample of the college students in one university located in Beijing, China. Two theoretical hypothesis were tested in the present study: (1) health risk perceptions differ as a function of demographics: age, gender, education and place of residence; hypothesis (2) health risk items correlated to each other and risks which are close in content tend to correlate more than risks which are distant in content. The findings of the present study will contribute to a better understanding of the health perception and health status profile of college students in China and will allow for the development of health-prevention programs for college students. Health risks involve technical understanding of health risk, public perceptions, and public influence. The perception of health risk will not only have a major influence on college students’ health behaviour, but also is an important consideration in health-prevention and programes. In order to develop effective health-prevention programes and health risk communication strategies, it is essential to have information about differences in opinion among colldge students.

## 2. Methods

### 2.1. Survey Content

The present survey was designed to investigate the perception of health risks among college students in China. Prior to its implementation, the study protocol was reviewed and approved by the Research Ethics Board of the University of Tsinghua.

Two questionnaires were used. The questionnaire begins with some brief demographics of the respondents, covering their age, gender, place of residence, and family background. The first questionnaire used consisted of three parts: background knowledge, health risks, source for health risk information. It begins with one common definition for the term health risk. Here, health risk is defined by the probability of the occurrence of a particular disease or the potential of a particular disease leading to death. The items of health risks were taken from Dake *et al.*’s 1991 risk questionnaire [33]. The risks that did not exist at the university were removed (e.g., high school football) and newer risks (global warming, GM foods) never before examined were added. These modifications produced a final list of 15 risks that ranged in area of effect from global, e.g., global warming or air pollution to local, e.g., cigarette smoking or drinking alcohol. Items are randomly listed for each respondent to avoid bias associated with the sequences of issues: (1) Global warming (GW), (2) Natural catastrophes (NC), (3) Nuclear power stations (NPS), (4) Thermal power plants (TPP), (5) Ozone hole (OH), (6) Air pollution (AP), (7) Chemical pollution (CP), (8) Pesticides in food (PIF), (9) Genetically modified foods (GMF), (10) Cigarette smoking (CS), (11) Drinking alcohol (DA), (12) Medical X-rays (MX), (13) Motor vehicle accidents (MVA), (14) Mental stress (MS), (15) Overtime study or work (OSOW). Most of the issues addressed in the survey are matters of general concern to Chinese college students. These health risks all appeared to fall into one of the three clusters: health-related environmental risk (GW, NC, OH, AP, CP, PIF), health-related technology risks (NPS, TPP, MX, GMF), and health-related social risks (CS, DA, OSOW, MS, MVC). Each respondent was asked to indicate his or her opinion about the health risks of these fifteen hazards to Chinese college students as a whole. The respondents were asked to provide their response based on a four point ordinal Likert scale: almost no health risk (d1 = 1, assignment = 0), slight health risk (d2 = 2, assignment = 33.33), moderate health risk (d3 = 3, assignment = 66.67), and high health risk (d4 = 4, assignment = 100).

In the first questionnaire, the respondents were also asked to indicate their sources for health risk information: (1) news media; (2) medical doctors; (3) extracurricular publications; (4) the Internet; (5) friends and relatives; (6) science fiction; (7) other. The amount of information about health risk issues from these seven different sources was measured by a 4-point scale: no information, a little information, a fair amount of information, a lot of information.

The second questionnaire was the International Physical Activity Questionnaire-short form [34]. The respondents were asked to indicate their physical activities over the last 7 days. The survey was also designed to account for the broad determinants of population health, including social and behavioral determinants, genetic and biological determinants, and environmental and occupational determinants, as well as health services and policies. Although multiple topics were surveyed, the results from these survey components will be reported separately. Only the sections of importance to the current study are presented here.

### 2.2. Survey Design and Implementation

A population-based study was administered during September and October 2012 at a university which is a major research university in China. The health risk perception was assessed through a questionnaire survey among college students. The students were told by their own teachers one week before the survey that responses would be anonymous and confidential, and written informed consent was obtained from each participant when they received the questionnaires. They were free to choose to participate in the present survey or choose not to fill out the questionnaires. All of the freshmen in Tsinghua University were selected as our study sample. All of the freshmen are required to participate in courses on physical education theory. The questionnaires were administered during 18 physical education theory classes on 15–19 October 2012. The questionnaires were given to college students by their teachers and by volunteers. No time limit was imposed. The students were asked to submit the survey at the end of the class. There was also the option to not answer and leave the classroom.

The completion of the questionnaire is a somewhat tedious task, and we sought to keep the amount of time necessary to complete the survey under 30 min. The content-related validity of the survey was ensured via systematic evaluations of the survey questions by the research team. Three pre-tests with volunteers were also conducted during 19–28 September 2012, to determine whether the questions were understood and to evaluate other formatting details.

Three samples of college students were chosen for the initial exploratory study. These samples all come from the same university. The first sample was 20 (seven male and 13 female) college students who were enrolled in a social science experiment class and completed the survey in their dormitory. The second sample was of 18 (six male and 12 female) college students who were enrolled in a humanities experiment class and also completed the survey in their dormitory. The third sample was of 19 (eight male and 11 female) college students who were all members of technology philosophy courses, and they completed the test in the classroom. In the first pre-test, six of the 20 participants raised questions about the meaning of the survey items. In the second pre-test, two of the 18 participants spent over 30 min completing the questionnaire. During the pretest phase, a number of meetings were held to discuss the risk list from Dake’s questionnaire and the translation version of the IPAQ. Adjustments were made to the survey tool following the pre-test; some items (high school football, commercial air travel) were removed, and some changes were made in the wording of questions. In the third pre-test, no questions were raised and all of the 19 participants complete the questionnaire in under 30 min.

A total of 3,105 individuals participated in the study. Of these surveys, 36 were invalid. The total number of valid samples is 3,069, among which 2,052 are male and 1,017 are female, representing 66.86% and 33.14% of the total, respectively. Regarding the students’ home residence, among the males, 268 are from municipalities directly under the central government, 338 are from the capital cities of provinces, 1,037 from are other cities and 409 are from towns and rural areas. Among the females, 204 are from municipalities directly under the central government, 199 are from the capital cities of provinces, 526 are from other cities and 88 are from counties and towns. The demographic data are presented in Table 1.

**Table 1:** Demographic data for participants.

	Municipalities	Capital cities	Counties & cities	Suburban & Rural	Total
**Male**	268	338	1,037	409	2,052
**Female**	204	199	526	88	1,017
**Total**	472	537	1,563	497	3,069

The lower female participation (33.14 percent) is due to the sex ratio of Tsinghua University. The total number of male students is almost two times the total number of female students in Tsinghua. The unequal gender distribution is due to the unequal distribution of subjects at Tsinghua University which is reknowned among universities in China for its focus on science and technology and its subjects in the field of Humanities and Social Sciences are relatively weak. The sample totally matched the gender distribution of students in Tsinghua University. Although not representative of the general population in China, a survey of college students offers some insight into the attitudes and concerns among the younger, more educated population; these students may have a greater awareness of the health risk issues related to science and technology than the general population.

### 2.3. Statistical Analysis

Descriptive statistics were used to illustrate the levels of risk perceived according to the percent of “high risk” responses as well as the mean response values (positive responses only). Chi-square tests were used to examine the group differences in risk perceived for each hazard according to the proportion of respondents perceiving a risk as “high”. The data were collected and analyzed with aid of SPSS Version 17.0 (IBM Corporation, Somers, NY, USA) for both descriptive statistics and correlation analysis. The differences in health risk perception will be shown through a comparison of the different issues, gender differences, and geographic differences. The significant differences will be the focus of this article.

## 3. Results

### 3.1. Perception of Health Risks to College Students in China

The perceived health risks to college students in China for the 15 hazards considered here are shown in Table 2, with the hazards ranked according to the percentage of the respondents who indicated that they considered it to be a “high health risk”.

Overall, traditional environmental risks such as natural catastrophes, pollution issues (chemical pollution and air pollution), and pesticides in food were ranked as relatively high risk. The respondents were less concerned about new emerging issues and long-term environmental risks (global warming). Health-related technology risks received the lowest percentage of “high health risk” responses, followed by GMF, medical X-rays, thermal power plants, and nuclear power stations. The risks related to the social environment including motor vehicle accidents and cigarette smoking were perceived to pose the greatest health risk.

**Table 2:** Health risk perceptions among Chinese college students.

N	Risk issue	Risk perception	Percent	Total		
				Medians	Std. Deviation	Chi-Square
**13**	**Motor vehicle accidents**	High risk	74.1	100.00	20.818	1.99E5 (a)
**7**	**Chemical pollution**	High risk	69.3	100.00	19.870	1.96E5 (a)
**10**	**Cigarette smoking**	High risk	67.0	100.00	21.595	1.59E5 (a)
**2**	**Natural catastrophes**	High risk	62.6	100.00	26.886	1.71E5 (a)
**8**	**Pesticides in food**	High risk	49.4	66.67	23.851	9.78E4 (a)
**6**	**Air pollution**	High risk	44.9	66.67	23.327	9.91E4 (a)
**5**	**Ozone hole**	High risk	42.0	66.67	25.603	1.02E5 (a)
**14**	**Mental stress**	High risk	37.6	66.67	26.499	6.58E4 (a)
**11**	**Drinking alcohol**	High risk	30.5	66.67	28.452	3.93E4 (a)
**3**	**Nuclear power stations**	High risk	30.2	66.67	32.128	3.29E4 (a)
**1**	**Global warming**	High risk	18.2	33.33	31.061	1.13E3 (a)
**12**	**Medical X-ray**	High risk	18.2	33.33	28.927	5.09E4 (a)
**15**	**Overtime study or work**	High risk	17.9	66.67	27.102	4.88E4 (a)
**4**	**Thermal power plants**	High risk	14.0	66.67	26.685	5.77E4 (a)
**9**	**GM foods**	High risk	11.2	33.33	29.335	4.74E4 (a)

a = Chi-square significant (*p* < 0.001).

In this survey, motor vehicle accidents (which are presumably felt to be beyond the victim’s control) were considered to be a “high health risk” by the greatest percentage of respondents. Chemical pollution, cigarette smoking, and natural catastrophes, in that order, received the next greatest percentage of “high health risk” responses.

Emerging health issues such as genetically modified foods and global warming were perceived to be moderately low risks. Genetically modified foods received the lowest percentage of “high health risk” responses, followed by thermal power, overtime study or work, medical X-rays, and global warming, respectively.

After we assigned values to the different choices, the mean score of each item was computed. The mean score of GM (1) is 48.80, NC (2): 81.61, NPP(3): 59.99, TP(4): 53.39, OH (5): 74.18, AP (6): 77.23, CP (7): 87.81, PIF (8): 78.61, GMF (9): 41.77, CS (10): 86.22, DA (11): 64.99, MXR (12): 51.95, MVA (13): 88.87, MS (14): 71.51, OSOW (15): 56.52. The ranking of these 15 items in sequence is (14), (4), (10), (12), (7), (6), (2), (5), (15), (3), (9), (13), (1), (8), (11). Overall, the items with the lowest means (<50) were global warming and genetically modified foods; the items with the highest means (>80) were motor vehicle accidents, chemical pollution, cigarette smoking and natural catastrophes. The degree of the respondents’ perception of health risks in the other items is moderate, which is between “2 = low risk” and “3 = medium risk”.

### 3.2. Demographic Health Risks

To investigate the links between risk perception and demographics, we must know which demographic characteristics are important for our survey. In risk perception studies, four factors linked to the perceiver are generally considered to be important variables: age, gender, education level, and region. According to previous studies, very few notable differences were detected between different age groups (18–25, 33–40, 26–32, above 40), and the level of education tends to be only weakly related to risk perception.

The distinctive character of the sample in our survey is that the age difference was so small (in the age range 16–25, so the subjects could all be assigned to the same age group), and the education level is the same (all college students). With regard to the character of the present sample, we consider the meaningful demographics in the present study to be gender and region.

The percent perceiving high risk for the selected hazards by demographic distribution is presented in Table 3. The mean value and standard deviation of each health risk perception for the demographic distribution are presented in Table 4.

*Gender difference.* As we can see from Table 3, the women perceived the risk associated with every hazard to be higher than the men did, with the exception of the differences between women and men in the percent assessing a “high health risk” for cigarette smoking, global warming, and thermal power plants. The greatest gender difference (13.10%) in the perceived risk in the present survey was observed for nuclear power stations. Other hazards in which large differences between men and women were noted were environmental and included natural catastrophes and chemical pollution. Actually, all gender differences were statistically significant (*p* < 0.001).

**Table 3:** Percent perceiving high health risk for selected hazards by demographic distribution.

N	Risk issue	Percent	Gender			Geographic Distribution				
			Male	Female	Chi-Square	Municip-alities	Capital cities	Counties & cities	Suburban & Rural	Chi-Square
**1**	**GW**	High risk	18.5	17.6	7.11E3 (a)	16.7	18.8	18.1	19.4	1.97E4 (a)
**2**	**NC**	High risk	59.3	69.3	1.33E4 (a)	61.8	59.6	63.0	66.5	6.98E4 (a)
**3**	**NPP**	High risk	25.9	39.0	1.85E3 (a)	26.4	31.8	30.1	31.4	3.38E4 (a)
**4**	**TP**	High risk	14.2	13.6	5.31E3 (a)	13.5	15.0	13.6	14.4	1.40E4 (a)
**5**	**OH**	High risk	41.5	43.0	2.27E4 (a)	37.3	41.9	41.8	47.5	8.14E4 (a)
**6**	**AP**	High risk	44.1	46.6	1.32E4 (a)	44.4	46.6	44.8	44.0	4.92E4 (a)
**7**	**CP**	High risk	66.4	75.0	1.65E4 (a)	65.7	70.4	68.9	72.3	7.52E4 (a)
**8**	**PIF**	High risk	48.0	52.1	2.51E4 (a)	44.8	50.3	50.2	51.3	8.87E4 (a)
**9**	**GMF**	High risk	10.9	11.9	3.02E3 (a)	10.9	12.6	11.2	9.40	1.29E4 (a)
**10**	**CS**	High risk	69.2	62.6	2.94E4 (a)	71.9	67.2	67.2	61.5	7.51E4 (a)
**11**	**DA**	High risk	29.2	33.3	3.15E4 (a)	31.5	33.0	30.9	26.3	9.64E4 (a)
**12**	**MXR**	High risk	16.1	22.3	1.80E3 (a)	16.7	19.5	19.2	14.6	2.51E4 (a)
**13**	**MVA**	High risk	72.9	76.5	2.19E4 (a)	70.1	76.5	74.6	75.4	8.31E4 (a)
**14**	**MS**	High risk	35.7	41.2	8.39E3 (a)	41.8	40.3	37.2	32.3	4.01E4 (a)
**15**	**OSOW**	High risk	18.5	16.7	7.96E3 (a)	20.0	18.4	17.2	17.6	1.66E4 (a)

Perceived health risk to Chinese college students: Difference by gender and region. a = Chi-square significant (*p* < 0.001).

**Table 4:** Mean value and standard deviation of each health risk perception for the demographic distribution.

Risk issue	Gender					Geographical Distribution								
	Male		Female		Chi-Square	Municipalities		Capital cities		Counties & cities		Suburban & Rural		Chi-Square
	Medians	Std. Deviation	Medians	Std. Deviation		Medians	Std. Deviation	Medians	Std. Deviation	Medians	Std. Deviation	Medians	Std. Deviation	
**GM**	33.33	32.115	33.33	28.311	1.27E4 (a)	33.33	30.684	33.33	31.704	33.33	30.777	33.33	31.177	5.61E4 (a)
**NC**	100.00	28.262	100.00	23.175	2.22E4 (a)	100.00	26.634	100.00	26.972	100.00	27.317	100.00	25.136	8.82E4 (a)
**NPS**	66.67	32.547	66.67	29.423	1.01E4 (a)	66.67	32.362	66.67	32.691	66.67	32.408	66.67	30.551	6.52E4 (a)
**TPP**	66.67	27.152	66.67	25.345	1.58E4 (a)	33.33	28.385	66.67	27.042	66.67	25.945	66.67	26.127	5.94E4 (a)
**OH**	66.67	26.375	66.67	23.458	2.27E4 (a)	66.67	26.160	66.67	26.215	66.67	25.350	66.67	24.124	8.14E4 (a)
**AP**	66.67	23.775	66.67	22.203	2.46E4 (a)	66.67	23.464	66.67	24.299	66.67	22.817	66.67	23.133	8.64E4 (a)
**CP**	100.00	20.474	100.00	18.024	2.68E4 (a)	100.00	22.839	100.00	19.993	100.00	19.173	100.00	17.957	9.76E4 (a)
**PIF**	66.67	24.143	100.00	22.874	2.51E4 (a)	66.67	25.157	100.00	23.487	100.00	23.319	100.00	23.739	8.87E4 (a)
**GMF**	33.33	29.797	33.33	27.557	7.89E3 (a)	33.33	29.965	33.33	29.474	33.33	29.301	33.33	28.231	4.43E4 (a)
**CS**	100.00	21.662	100.00	21.340	3.15E4 (a)	100.00	20.954	100.00	21.772	100.00	21.068	100.00	23.090	9.64E4 (a)
**DA**	66.67	29.028	66.67	28.790	1.80E4 (a)	66.67	30.067	66.67	29.825	66.67	27.844	66.67	27.316	7.50E4 (a)
**MXR**	33.33	28.415	66.67	29.181	1.20E4 (a)	33.33	29.129	33.33	28.918	33.33	29.329	33.33	26.766	5.71E4 (a)
**MVC**	100.00	21.571	100.00	18.700	2.80E4 (a)	100.00	22.632	100.00	19.537	100.00	20.705	100.00	18.866	9.84E4 (a)
**MP**	66.67	27.130	66.67	24.831	2.08E4 (a)	66.67	27.447	66.67	27.470	66.67	26.188	66.67	25.458	7.93E4 (a)
**OSOW**	66.67	27.704	66.67	25.803	1.90E4 (a)	66.67	28.161	66.67	27.547	66.67	27.012	66.67	25.803	6.06E4 (a)

Perceived health risk to Chinese college students: Difference by gender and region. a = Chi-square significant (*p* < 0.001).

An evaluation of the mean scores by gender revealed similar results, with the mean score assigned by women exceeding that for men (with the sole exception of cigarette smoking; the results are shown in Table 4.

*Geographic differences.* Table 3 shows the differences in perceived “high health risk” by geographic regions in those cases where significant differences among regions were observed (*p* < 0.001). With the hazards ranked according to the percentage of respondents that they considered the hazard to be a “high health risk”, the respondents in municipalities were significantly more likely to rate a number of health items to be a lower risk than did respondents in the other regions. We also observed some exceptions in the differences: the respondents in suburban and rural areas compared to the other regions tend to rate health risks such as cigarette smoking, drinking alcohol, and mental stress to be lower. The greatest *geographic* difference (10.40%) in perceived risk in the present survey was observed for cigarette smoking, followed by the ozone hole (10.20%) and mental stress (9.50%). An evaluation of the mean scores by region revealed similar results, and the results are shown in Table 4.

### 3.3. Association between Different Health Risk Perceptions

It is a general and powerful principle that risks which are close in content tend to correlate more than risks which are distant in content. A further analysis of the environmental risk, technological risk and social risk was of interest. To investigate the relationship between different risk perceptions, two-tailed tests were used to calculate the correlations between each of the items. As shown in Table 5, most of the correlations were statistically significant. A total of 225 correlations were listed in Table 5, and 209 of these were significant, indicating that the degree of the participants’ health risk perception towards the listed items was correlative to some extent. The highest correlation was 0.571. In addition, there were very few negative correlations: the highest negative correlation was 0.018 (and was not statistically significant).

The perception of the participants towards residual insecticide in food was closely associated with chemical pollution (the maximum correlation of r = 0.571, moderate positive correlation) and was statistically significant at the 0.01 level (2-tailed). In addition, the correlations between thermal power and nuclear energy power, the ozone hole and thermal power, air pollution and thermal power, air pollution and the ozone hole, chemical pollution and the ozone hole, chemical pollution and air pollution, residual insecticides in food and air pollution, drinking and smoking, motor vehicle accidents and natural disasters, and overtime working/learning and mental stress appear to be statistically significant (0.3 < r < 0.5, moderate positive correlation) as well according to the participants’ perception reports. The correlations between most of the other items were weak (r < 0.3).

Overall, the present survey yielded high correlations between environmental risks (the ozone hole, air pollution, chemical pollution, pesticides in food) and technological risks (nuclear power stations, thermal power plants). The items belonging to social health risks (cigarette smoking and drinking alcohol, overtime study or work and mental stress) are also strongly correlated.

**Table 5:** Relationship between each health risk issue.

	GM	NC	NPS	TPP	OH	AP	CP	PIF	GMF	CS	DA	MXR	MVC	MP	OSOW
**GM**	1.000	0.279^ **^	0.194 **	0.253 **	0.391 **	0.161 **	0.117 **	0.087 **	0.200 **	0.005	−0.005	0.078 **	−0.006	0.116 **	0.077 **
**NC**	0.279 **	1.000	0.215 **	0.168 **	0.225 **	0.105 **	0.209 **	0.154 **	0.103 **	0.014	0.011	0.078 **	0.322 **	0.067 **	0.027
**NPS**	0.194 **	0.215 **	1.000	0.322 **	0.282 **	0.116 **	0.180 **	0.141 **	0.259 **	−0.018	0.028	0.256 **	0.107 **	0.063 **	0.060 **
**TPP**	0.253 **	0.168 **	0.322 **	1.000	0.444 **	0.344 **	0.268 **	0.226 **	0.220 **	0.148 **	0.124 **	0.178 **	0.114 **	0.158 **	0.160 **
**OH**	0.391 **	0.225 **	0.282 **	0.444 **	1.000	0.455 **	0.345 **	0.296 **	0.211 **	0.150 **	0.081 **	0.164 **	0.072 **	0.185 **	0.142 **
**AP**	0.161 **	0.105 **	0.116 **	0.344 **	0.455 **	1.000	0.485 **	0.471 **	0.211 **	0.296 **	0.188 **	0.167 **	0.114 **	0.238 **	0.197 **
**CP**	0.117 **	0.209 **	0.180 **	0.268 **	0.345 **	0.485 **	1.000	0.571 **	0.179 **	0.236 **	0.134 **	0.189 **	0.240 **	0.172 **	0.135 **
**PIF**	0.087 **	0.154 **	0.141 **	0.226 **	0.296 **	0.471 **	0.571 **	1.000	0.240 **	0.271 **	0.178 **	0.231 **	0.206 **	0.180 **	0.174 **
**GMF**	0.200 **	0.103 **	0.259 **	0.220 **	0.211 **	0.211 **	0.179 **	0.240 **	1.000	0.096 **	0.160 **	0.259 **	0.072 **	0.135 **	0.150 **
**CS**	0.005	0.014	−0.018	0.148 **	0.150 **	0.296 **	0.236 **	0.271 **	0.096 **	1.000	0.471 **	0.131 **	0.153 **	0.164 **	0.149 **
**DA**	−0.005	0.011	0.028	0.124 **	0.081 **	0.188 **	0.134 **	0.178 **	0.160 **	0.471 **	1.000	0.181 **	0.144 **	0.147 **	0.142 **
**MXR**	0.078 **	0.078 **	0.256 **	0.178 **	0.164 **	0.167 **	0.189 **	0.231 **	0.259 **	0.131 **	0.181 **	1.000	0.193 **	0.121 **	0.132 **
**MVC**	−0.006	0.322 **	0.107 **	0.114 **	0.072 **	0.114 **	0.240 **	0.206 **	0.072 **	0.153 **	0.144 **	0.193 **	1.000	0.140 **	0.083 **
**MP**	0.116 **	0.067 **	0.063 **	0.158 **	0.185 **	0.238 **	0.172 **	0.180 **	0.135 **	0.164 **	0.147 **	0.121 **	0.140 **	1.000	0.452 **
**OSOW**	0.077 **	0.027	0.060 **	0.160 **	0.142 **	0.197 **	0.135 **	0.174 **	0.150 **	0.149 **	0.142 **	0.132 **	0.083 **	0.452 **	1.000

** Correlation is significant at the 0.01 level (2-tailed).

## 4. Discussion

In this study, the general order of risk perception was consistent between the males and females; however, the females were more likely to rank items as a higher risk than the males. This finding is in agreement with previous studies, such as that of Flynn *et al.* [35] and of Samil [36]. This result suggests that the instrument used is of value for measuring Chinese college students’ perceptions of health risks. Of course, further study should be performed to test this result’s validity in other groups. Each of the studies has used a slightly different set of items, and the list of risks should also be modified according to different circumstance in the future.

Out of 3,105 questionnaires distributed, 3,101 respondents filled out the questionnaire, which resulted in a response rate of 99.8 per cent. The high-reponse rate can be interpreted by the following factors: the questionnaire distribution method (through one-to-one distribution), the questionnaire distribution place (in the classroom), the questionnaire distribution time (during physical education theory classes), the respondents (freshmen). The four factors all increased the chances of an individual’s participation to the questionnaire. Health issues is one important topic in physical education theory classes which is one required course for all freshmen in China. Tsinghua University is one of the leading universities in China, and its freshmen show their extreme concerns about health risks and their great enthusiasm for scientific research. Although there was also the option to not fill the questionnaire and leave the classroom, almost all of the students submitted the survey.

Most of the environmental health risks are related to the physical environment. In the present study, traditional environmental risks such as natural catastrophes, pollution issues (chemical pollution, air pollution), and pesticides in food were ranked as having relatively high risks. The respondents were less concerned about the new emerging issues and long-term environmental risks (global warming). The famous “map” of hazards in the three major dimensions (dread, novelty and catastrophic potential) can be used to explain how the Chinese college students ranked the issues related to environmental risks. The consequences of the traditional environmental risks can be catastrophic and involve a higher feeling of dread, so the college students tend to rank them as higher risks.

The Chinese college students ranked the items related to technology risks as having the lowest risks, followed by GMF, MXR, TPP, and NPS. This phenomenon is commonly found in risk perception surveys and has been referred as an “optimistic bias”. This bias may be explained by the fact that these technologies are critical for survival or the enhancement of their quality of life and the Chinese people have benefitted significantly from the development of Science & Technology since the implementation of reforms and the new policy of openness in 1978.

There is still little consensus regarding the relationship between health concerns in the general attitudes toward GMF [24]. Consumers in the United States appear to be the least concerned about the negative health effects from GMF, whereas the consumers in Europe and Asia appear to be more concerned [37]. Chinese consumers appear to be completely indifferent to or even to feel positively toward GMF [38]. Our findings revealed that the new emerging health issues such as GMF were perceived as having moderately low risks. GMF received the lowest percentage of “high health risk” responses in our survey. These results tell us that most of the college students in China are more present-oriented regarding health. These students perceive the risks of GMF as being the lowest of all risk issues considered, perhaps because young college students are the least likely to be concerned about risks that impact future health. The social health risk perceptions comprised high loadings on the items pertaining to lifestyle, as well as the items relating to the social environment. The social health risks (the behavioral and lifestyle factors) were ranked among the highest of all of the risk issues considered. Traffic deaths in China have been ranked first in the World for ten years; 6.2 million people died in car accidents in 2011. Health risk perception refers to the views or attitudes that people have of health risks. According to our survey, automobile accidents were ranked the highest of all of the risk issues considered among college students in China. This result may be explained by the following factors: MVA are presumably felt to be beyond the victim’s control; the news media predominate as an information source regarding health risk information and traffic accidents attract the media’s attention; and school counselors often prompt students, especially freshmen, to pay particular attention to traffic safety. Therefore, risk is not an objective entity: it may include physical, social and psychological aspects. Risk should not be measured independently of the context in which the hazards occur.

Previous studies [39,40,41,42] reported that only 1% of women aged 20 to 29 years were smokers while 63% of men in this same age group described themselves as current smokers in 1996. Although the entire Chinese society became more open and diverse, there has not been a significant increase in the smoking rate among females. Cigarette smoking is still a major public health problem in China. The level of risk that is perceived to be associated with a hazard depends on the degree to which the hazard is understood and whether it results in immediate or in delayed death. There is a need for better education about cigarette smoking, drinking alcohol, nuclear power stations and general health in universities. Universities may be falling short in their responsibility towards students if they do not make this knowledge available.

According to the cross tabulation analysis results, the perception of the health risks considered differs by gender and region. Consistent with the results from several studies [37,43,44,45,46], our study indicated that female college students in China are more concerned about health risks than male college students.

Cigarette smoking is a recognized cause of morbidity and premature mortality. Our survey found an exception in the gender differences for cigarette smoking: female college students perceive the risks to be lower than the male college students. This finding may be explained by the cultural circumstances of cigarette smoking behavior. Currently, tobacco companies target women with advertising. Associating smoking with slimness has been shown to encourage smoking initiation among adolescent girls and young women. Females also speak of the positive attributes of smoking a cigarette, for example, enhancing one’s sense of mystery and complexity and appearing free to take risks [41,47,48].

Considering the fact that the presence or absence of hazards often depends on geographic location, it is not surprising to find that the health risk perceptions differed by region. The regional differences in risk perception may be influenced by a number of factors, such as proximity to the hazard, sociopolitical climate and the nature and type of information sources available [49]. The respondents in municipalities were significantly less concerned about most of the health risks than the respondents in other regions. This result might be attributed to a number of complicated reasons. First, stronger economic development may lower risk perception. A second source of greater risk tolerance may stem from the high standard of living in municipalities that may promote general optimism and self-confidence regarding the future, which both have the effect of lowering peoples’ risk perceptions. A third factor in the lower risk tolerance rural areas may be the lack of sufficient information about potential risks [31]. The impact of information on forming risk perceptions can play an important role in the demand adjustment to unexpected shocks.

In the present study, we also found that the residents in small town or rural areas, compared to other regions, tend to rate such health risks as cigarette smoking, drinking alcohol, and mental stress to be lower. The greatest geographic difference (10.40%) in the perceived risk in the present survey was observed for cigarette smoking.

## 5. Summary and Conclusions

The present large-scale survey of the public perception of health risks among college students in China presents valuable information on their perception of health risks. Generally, the hazards that were perceived as posing the greatest health risk were those belonging to the social health risks category; items related to technology risks received the lowest percentage of “high health risk” rankings. Traditional environmental risks were ranked as being relatively high risks. The respondents were less concerned about new emerging issues and long-term environmental risks. The survey demonstrated higher perceptions of health risk among females as compared to males among college students in China. Most gender differences were statistically significant. Regional differences in risk perceptions were also observed in our survey. The respondents in municipalities were more likely to rate a number of the health items to be lower risks.

The limitations of this study should be elucidated. The present results have been limited to a description of how the hazards were rated in terms of health risk. Further studies are needed to increase our understanding of the influence of gender and region on health risk perceptions. Prospective studies among college students would be required to establish a causal link between health risk perceptions and health-related behavior. This study used a sampling of students from one University; therefore, generalizability to other settings or populations may also be limited. Even with these limitations, this study suggests that the unique characteristics and cultural factors related to health risk perception among college students in China warrant special consideration in the development and design of risk prevention programs.

The grouping of health risks into environmental, technological, and social risks may provide valuable insight into the intricate nature of relationships between health risk perceptions. Understanding how Chinese college students form attitudes about health risk can help in designing effective health risk communication programs. At a practical level, this acquired understanding could prove useful to guide the development of educational campaigns and policies on health. It would be valuable to examine whether the current findings on health risk perceptions among college students translate into lifestyle changes in future research.
